# Simplifying drug package leaflets written in Spanish by using word embedding

**DOI:** 10.1186/s13326-017-0156-7

**Published:** 2017-09-29

**Authors:** Isabel Segura-Bedmar, Paloma Martínez

**Affiliations:** Computer Science Departament, Universidad Carlos III de Madrid, Avenida de la Universidad, 30, Madrid, Spain

**Keywords:** Text simplification, Lexical simplification, Word embeddings, Drug package leaflets

## Abstract

**Background:**

Drug Package Leaflets (DPLs) provide information for patients on how to safely use medicines. Pharmaceutical companies are responsible for producing these documents. However, several studies have shown that patients usually have problems in understanding sections describing posology (dosage quantity and prescription), contraindications and adverse drug reactions. An ultimate goal of this work is to provide an automatic approach that helps these companies to write drug package leaflets in an easy-to-understand language. Natural language processing has become a powerful tool for improving patient care and advancing medicine because it leads to automatically process the large amount of unstructured information needed for patient care. However, to the best of our knowledge, no research has been done on the automatic simplification of drug package leaflets. In a previous work, we proposed to use domain terminological resources for gathering a set of synonyms for a given target term. A potential drawback of this approach is that it depends heavily on the existence of dictionaries, however these are not always available for any domain and language or if they exist, their coverage is very scarce. To overcome this limitation, we propose the use of word embeddings to identify the simplest synonym for a given term. Word embedding models represent each word in a corpus with a vector in a semantic space. Our approach is based on assumption that synonyms should have close vectors because they occur in similar contexts.

**Results:**

In our evaluation, we used the corpus EasyDPL (Easy Drug Package Leaflets), a collection of 306 leaflets written in Spanish and manually annotated with 1400 adverse drug effects and their simplest synonyms. We focus on leaflets written in Spanish because it is the second most widely spoken language on the world, but as for the existence of terminological resources, the Spanish language is usually less prolific than the English language. Our experiments show an accuracy of 38.5% using word embeddings.

**Conclusions:**

This work provides a promising approach to simplify DPLs without using terminological resources or parallel corpora. Moreover, it could be easily adapted to different domains and languages. However, more research efforts are needed to improve our approach based on word embedding because it does not overcome our previous work using dictionaries yet.

## Background

Since 2001, according to a directive of the European Parliament (Directive 2001/83/EC) [1], every drug product must be accompanied by a package leaflet before being placed on the market. This document provides informative details about a medicine, including its appearance, actions, side effects and drug interactions, contraindications, special warnings, among others. This directive also required that drug package leaflets (DPLs) must be written in order to provide clear and comprehensible information for patients because their misunderstanding could be a potential source of drug related problems, such as medication errors and adverse drug reactions.

In 2009, the European Commission published a guideline [2] with recommendations and advices in order to issue package leaflets with accessible and understandable information for patients. However, recent studies [3, 4] show that the readability and understandability of these documents have not been improved during the last years. In particular, a recent work [5] about readability of DPLs corresponding to 36 drugs downloaded from European Medicines Agency website in 2007, 2010 and 2013 years, concluded that there was no improvement in the readability of the package leaflets studied between 2007 and 2013, despite the European Commission’s 2009 guideline on the readability of these leaflets that established different rules to guarantee that patients can easily understand them. Therefore, further efforts must be made to improve the readability and understandability of DPLs in order to ensure the proper use of medicines and to increase patient safety.

One of the main reasons why the understandability has not been improved is that these documents still contain a considerable number of technical terms describing adverse drug reactions, diseases and other medical concepts. Posology (dosage quantity and prescription), contraindications and adverse drug reactions seem to be the sections most difficult to understand [6]. To help solving this problem, we propose an automatic system to simplify those terms describing adverse drug effects in DPLs. Text simplification is a natural language processing (NLP) task that aims to rewrite text into an equivalent one with less complexity for readers. Text simplification techniques have been applied to simplify texts from different domains such as crisis management [7], health information [8–10], aphasic readers [11], language learners [12].

To the best of our knowledge, our previous work [13] is the only research about the automatic simplification of DPLs. We focus on lexical simplification, that is, the substitution of complex concepts with simpler synonyms. Moreover, we focus on leaflets written in Spanish because it is the second most widely spoken language on the world^1^. Our first approach consisted of choosing the most frequent synonym as the simplest one. To do this, we used specialized dictionaries for medicine for obtaining the set of synonym candidates for a given term, and then, we calculated their frequencies in a large collection of documents. In this new work, we focus our efforts on exploring a domain-independent and language-independent approach, such as the use of word embedding. A word embedding is a function that transforms words into real-value vectors. This representation ensures that similar words have similar vectors, that is, their vectors are close together. At this time there is a explosion of research based on word embeddings applied to a wide variety of NLP tasks with very successful results. Although several works have already exploited the use of word embeddings for detecting complex words [14], building parallel corpus for text simplification [15] or substitution of complex words [16], the lexical simplification of DPLs is still an unexplored field. In addition, our work is one of the few studies that addresses the simplification of texts written in Spanish.

## Related work

There are two main subtasks of text simplification: lexical and syntactic simplification. Lexical simplification basically consists of replacing complex concepts with simpler synonyms, while syntactic simplification aims to reduce the grammatical complexity of a text while preserving its meaning. Comprehensive surveys of the text simplification field can be found in [17, 18]. We have to distinguish between readability and understandability because these concepts capture different aspects of the complexity of the text. Readability concerns the length and structure of sentences (syntax) and consequently requires syntactic simplification approaches to split sentences in shorter units with simpler structure. On the other side, understandability is about the difficulty to interpret a word [19] and it requires lexical simplification approaches. Our work here focuses on improving the understandability of DPLs by replacing terms describing drug effects by simpler synonyms.

The main challenges of lexical simplification are (a) the difficulty of recognizing if a word is a complex term and (b) identifying the correct synonym for a particular context in which the word appears (this is crucial, especially when the word is polysemous). For the first issue (a), a common heuristic used is to select as complex words those that have a low frequency in a corpus (complex words tend to be rarer), but also to combine frequency with word length (words with more than a number of syllables/characters could be considered complicated words). In Semeval 2012 English Lexical Simplification challenge^2^ with ten participant systems, the evaluation results showed that proposals based on frequency gave good results comparing to other sophisticated systems. Similarly, the complex word identification task of SemEval 2016 [20] showed that though decision trees and ensemble methods achieved satisfactory performance, word frequency is still the most efficient predictor of word complexity. Decision trees and ensemble methods perform better than neural networks because the small size of the training dataset. The only system exploiting word embeddings was developed by Sanjay et al. [14], who trained a support vector machine (SVM) algorithm to identify complex words. In addition to word embeddings, the feature set also included orthographic word features, similarity features and Part-of-Speech (POS) tag features.

Parallel corpora of original and simplified texts can be used for automatic text simplification. Biran et al. [21] used English Wikipedia and Simple English Wikipedia [22] (which was developed applying Easy-to-Read (E2R) guidelines^3^) to calculate the complexity of a word as the ratio of its frequencies in each corpus.

For the issue (b), there are two main approaches: using lexical resources or using context words and n-grams models. Lexical resources (such as WordNet [23]) are used to propose synonyms as candidates in order to replace complex wordS. Lexical resources are also combined with probabilistic models, as has been tried in [24]. In the second approach, word contexts are used in [25] and [21], where a vector space model is used to capture lexical semantics and context preferences of words.

Focusing on research devoted to synonym substitution in Spanish texts, lack of large coverage semantic resources is a fundamental handicap. English Wordnet includes approximately 187 K meanings while the Spanish portion of EuroWordNet, [26], includes about 50 K word meanings. LexSiS system, [27], uses the Spanish Open Thesaurus (a freely available dictionary with approximately 21 K lemmas and their corresponding word senses)^4^ to propose a set of substitution candidates for a target word. Additionally, a vector is built in a window of nine words around each word-sense in a corpus of 8M words extracted from the Web and compared using the cosine similarity (according to the distributional hypothesis that establishes that different uses of a word tend to appear in different lexical contexts); distance between vectors is used to discard not adequate substitutes extracted from OpenThesaurus. Word frequency and word length are linearly combined to select the simplest term. This linear combination is not trivial as is reported in [27]. This approach can be enhanced including rule-based lexical simplification [28], where some patterns that avoid incorrect substitutions are defined, for instance, to replace reporting verbs (confirm, suggest, explain, etc.) that leaves correct syntactic structures as well as other editing transformations (numerical expressions or periphrasis). Following the same approach, CASSA method is reported in [29] where the Spanish corpus used to extract word occurrences is the Google Books Ngram corpus [30] that contains real web frequencies. This work also obtains word senses from OpenThesaurus.

There are more recent approaches that cope with the lack of language dependant resources (dictionaries and annotated corpora) in order to assure applicability in low-resources languages. For instance, [31] proposes an unsupervised method based on word vector representations extracted from regular texts to find adequate simplifications for complex words. In a first step, GloVe [32], a global log bilinear regression model, is used to obtain vector representations from English Wikipedia and the English Gigaword Fifth Edition corpus^5^. For each content word (verbs, nouns, adverbs and adjectives), the top 10 most similar GloVe words vectors are selected as simplification candidates. Then, these candidates are ranked taking into account semantic similarity using GloVe vectors and context similarity to avoid selecting a synonym of a wrong sense instead of the correct one. The hypothesis is that synonyms of the correct sense of a word are semantically closer to the context of this word. There are two other features considered in this ranking: comparing the information content of the original word and the simplification candidate calculated using frequencies extracted from Google Book Ngrams corpus and language model features that measure if a candidate fits into the sequence of words that precedes and follows the original word. This work has been evaluated using a crowdsourced dataset where manual simplifications have been proposed by 50 people and on the SemEval 2012 lexical simplification task for English [33]; results showed that this approach outperforms previous systems, such as [21]. However, this approach has an important limitation: word embeddings are not able to distinguish between all possible senses of a polysemous word. To overcome this limitation of the word embeddings models, Paetzold and Specia [16] added two constraints to select the synonym candidates: the candidate and target word must have the same POS tag, and they must not have the same stem. This work outperforms state-of-the-art work in Lexical Simplification.

## Methods

In this section, we describe our system in more detail. In our previous work, we combined a dictionary-based approach to give a set of synonyms for a given term, and then, to obtain their frequencies from a large collection of texts in order to propose the most frequent synonym as the simplest one. The novelty of this paper consists in using word embedding for finding the simplest synonym for a given term. This novel approach overcomes the limitations of the previous work because it does not depend on the existence of any dictionary of synonyms for a given domain and for a given language.

To evaluate our approach, we use the corpus EasyDPL (Easy Drug Package Leaflets), [13]. This corpus consists of 306 leaflets written in Spanish and manually annotated with 1400 adverse drug effects and their simplest synonyms.

**Fig. 1:**
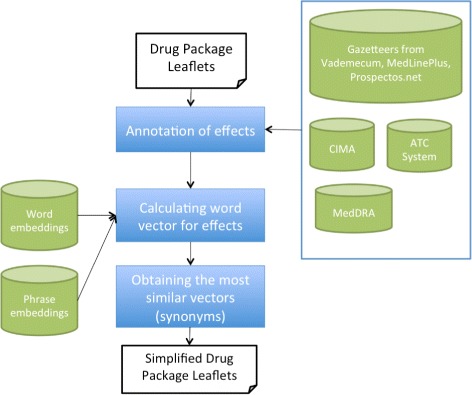
Overall system architecture

As illustrated in Fig. 1, the overall architecture of our system comprises three separate components. Briefly, first, the leaflets are processed and their adverse drug effects are annotated using a dictionary-based approach. Second, for each identified effect, we obtain its vector from a pre-trained word embedding model. In a word embedding model, similar meanings usually have similar vectors. Therefore, we use this model to obtain the most similar vectors for a given term. In the following subsections we describe in detail each of the previous tasks.

### Recognizing adverse drug effects

In our study, we focus on the simplification of adverse drug effects because evidence shows that patients often misinterpret or do not understand much of the information written in the section describing these effects. Therefore, the first task that we have to solve is the recognition of adverse drug effects in texts. To do this, we develop a NER (named entity recognition) module based on a dictionary-based approach that combines terminological resources such as the ATC system (a drug classification system developed by the World Health Organization), CIMA^6^ (a database that contains information on all drugs authorized in Spain, with a total of 16,418 brand drugs and 2,228 generic drugs) and several dictionaries gathered from websites about health and medicines such as MedlinePlus^7^, vademecum.es^8^ or prospectos.net^9^. Among the different resources used by the NER module, the MedDRA dictionary ^10^ stands out for its broad coverage of events associated with drugs. The main advantage of MedDRA is that its structured format allows easily obtaining a list of possible drug effects and their synonyms. MedDRA is composed of a five-level hierarchy. The most specific level, “Lowest Level Terms” (LLTs)”, contains a total of 72,072 terms that express how information is communicated in practice. Another important online resource for the NER module is MedlinePlus. It provides health information for patients, which contains more than 1000 articles about diseases and 6000 articles about medicines. The Spanish version is one of the most comprehensive and trusted Spanish language health websites at the moment. We developed a web crawler to browse and download pages related to drugs and diseases from its website. Each MedlinePlus article provides exhaustive information about a given medical concept, and also proposes a list of health-related topics, which can be considered as synonyms of this concept. Moreover, an article related to a given medical concept could be used to obtain the definition of this concept by getting its first sentence. The reader can find a detailed description of the NER module in [34].

Once we have already detected adverse drug effects in text, we can continue with the lexical simplification of these terms. We start describing our baseline approach based on dictionaries. Then, we describe our approach using word embedding.

### Generating synonynm candidates

As mentioned above, our goal aims to simplify DPLs, in particular, replacing the terms describing adverse drug effects with synonyms that are easier to understand for patients. Once adverse drug effects are automatically identified in texts, the following step is to propose a set of synonyms for each one of them.

An important drawback of our previous work is that it required dictionaries that provide a set of synonym candidates for a given word. To remedy this, we employ Word2Vec [35], a predictive model for learning word embeddings from raw texts. In particular, this model represents each word in a corpus as a vector in a semantic space. Thus, it is possible to compute the similarity of two words by calculating the cosine of the angle between their corresponding word vectors.

To obtain our synonym candidates, we use Cardellino’s pre-trained model [36], which is available for research community and was built from several Spanish collection texts such as Spanish Wikipedia (2015), the OPUS corpora [37] or the Ancora corpus [38], among others. It contains nearly 1.5 billion words and the dimension of its word vectors is 300.

The simplest approach could be to select the synonym candidate with the highest semantic similarity to the original word, however, this approach may not work for polysemous words. As is well known, the context in which a word occurs plays a central role to identify the sense of this word [39, 40]. Because word embeddings are able to capture the semantic similarity between words based on their contexts, our hypothesis is that the best synonym candidate should also be semantically similar to the words that occur around of the original word. Therefore, we do not only consider the semantic similarity between the synonym candidate and the original word, but also between the synonym candidate and the context words of the original word. To calculate the semantic similarity between a synonym candidate and the context words of the original word to be simplified, we compute the average of the cosine distance between all context words and the synonym candidate, as was proposed in [31]:

1 $$ csim(s,w)= \frac{1}{|C(w)|}\sum\limits_{w^{\prime} \in C(w)}cos(v_{s},v_{w^{\prime}})  $$

where s is the synonym candidate, w is the original word to be simplified, C(w) is set of the context words of w (we use a window of size three around of w) and *v*_*x*_ refers to the word vector of a word x.

In some cases, the word to simplify could be very simple, and therefore, it would not be necessary to replace it by any synonym. For example, adverse drug effects such as *dolor de cabeza (headache), depresión (depression) or vómitos (gastric juices)* are already very easy to understand and it is not necessary to replace them. Indeed, though the word embedding model is capable of proposing a set of synonyms for at least 72% of the adverse drug effects present in the EasyDPL corpus, these candidates are not always simpler than the original effect. Therefore, our system should be able to distinguish when a candidate is simpler than the original word. Based on the work performed by Devlin and Unthank [41], the complexity of a word seems to be directly related to its degree of informativeness. In other words, the more informative a word is, the more complex it tends to be. To measure the degree of informative of a word, we use the function defined in [41] and showed below:

2 $$ ci(w)= -log\left(\frac{freq(w)+1}{{\sum\nolimits}_{w^{\prime} \in C}freq(w^{\prime})+1}\right)  $$

where freq(w) is the frequency of the word w in a collection of texts C. Thus, using this function, the system replaces an original word by one of its candidates only if the candidate is less informative than the original word. To obtain the frequencies of the words, we use the Spanish version of the Google Book Ngram corpus [30]. In the EasyDPL corpus, over 51% of the adverse drug effects have at least a candidate less informative than them.

In a preliminary evaluation of our system, we noted that many errors were due to over 48% of the gold standards synonyms proposed in the EasyDPL are compound names.

For example, the gold synonym for the effect *anorexia* (anorexy) is the noun phrase *trastornos de la alimentación* (eating disorders). Another example is *acatisia* (akathisia), whose gold standard synonym proposed in the EasyDPL corpus is *incapacidad de quedarse quieto* (inability to stand still). Our approach cannot propose multi-word candidates because it is based on a word embedding model that only calculates the semantic similarity between vectors of tokens. To overcome this problem, we propose a simple approach to obtain phrase embeddings. This approach consists of applying a set of patterns based on POS tags to detect noun phrases describing adverse drug effects. Some of these patterns are shown bellow:

- NN ADJ. This pattern lets to recognize adverse drug effects such as *sueño anormal* (abnormal dream).
- NN PREP NN. It lets to identify adverse drug effects such as *enfermedad del estómago* (stomach disease).
- NN PREP VB. It detects adverse drug effects such as *problemas para tragar* (difficulty swallowing).
- NN ADJ PREP NN. This identifies adverse drug effects such as *azucar alta en sangre* (high blood glucose).

These patterns could recognize a huge number of noun phrases that are not actually adverse drug effects. To reduce this noise, we only consider some noun phrases that contain at least a word belonging to the MedDRA dictionary. To obtain our set of phrase candidates for adverse drug effects, we process our collection of downloaded MedLinePlus articles. POS tagging was performed using the Python NLTK4 POS-tagger^11^ adapted to Spanish language. We gather a total of 3000 phrase candidates that could describe adverse drug effects. Then, for each of these phrases, we obtain a phrase embedding by averaging the word embedding vectors of their content words (nouns, lexical verbs, adjectives and adverbs). Therefore, when we obtain the synonym candidate for a given adverse drug effect, we do not only consider the most similar word embeddings from single words, but also calculate the semantic similarity between the original effect and all phrases collected from MedLinePlus.

## Results

As it was mentioned above, the dataset used for the evaluation is the EasyDPL corpus consisting of 306 package leaflets manually annotated with 1400 adverse drug effects and their simplest synonyms. For each drug effect annotated in the EasyDPL corpus, the evaluation consisted in comparing the gold-standard synonym, that is, the synonym proposed by the human annotators, to the synonym candidate proposed by the system. Table 1 shows the results of our different approaches.

**Table 1:** Results

Approach	Accuracy
MedDRA	0.372
MedLinePlus	0.687
Word Embeddings	0.368
Word + Phrase Embeddings	0.385

## Discussion

In this section, we discuss some results from our experiments. Firstly, we focus on the results of our previous work based on dictionaries, which is also considered as the baseline system. For the synonym obtained from MedlinePlus, the baseline system achieved an accuracy of 68.7%, while for the MedDRA synonym, the accuracy is much lower (around 37.2%). This is mainly due to MedDRA being a highly specific standardized medical terminology, which implies its terms are not familiar to most people. MedlinePlus on the other hand is a health information website for patients, which uses a more readable language and a lay vocabulary.

As it is well known inter-annotator agreement (IAA) determines the complexity of the task and provides an upper bound on the performance of the automatic systems. For the EasyDPL corpus, the Fleiss’ kappa [42] was calculated, which is an extension of Cohen’s kappa [43]. This metric lets to measure the degree of consistency for two or more annotators. The assessment showed a kappa of 0.709, which is considered substantial on the Landis and Koch scale [44]. Therefore, we can conclude that the baseline system using MedlinePlus achieves results (68.7%) very close to those ones provided by the humans.

The approach based on word embeddings achieves an accuracy of 36.85%, slightly lower than the baseline system using the MedDRA dictionary, and significant lower than that obtained with the synonyms gathered from MedlinePlus. A main limitation of this approach is that it can only propose uni-word synonyms because the set of synonym candidates is obtained from a word embedding model. However, over 48% of the effects annotated in the EasyDPL corpus have a multi-word synonym. Thus, if we only evaluate the approach on those effects whose their gold synonyms are uni-words, the accuracy increases to 47.8%. We also evaluate our approach using word and phrase embbedings. In this case, the accuracy is 38.5%, which represents an improvement of 2% over the baseline system using MedDRA.

We conducted an error analysis in order to obtain the main causes of errors in our system. In particular, we studied in detail a random sample of 30 documents. The error analysis also showed that some of the synonyms proposed by the system might be right answers, even though they are not the same as proposed by the EasyDPL corpus. Most errors are due to the gold synonym for a given effect could be a long and complex noun phrase or even a small sentence. For example, the gold standard synonym for the effect *agranulocitosis* is *poca producción de defensas en la sangre* (poor production of defences in the blood). Our patterns proposed to detect phrases are not able to identify theses sentences as candidates. Moreover, it should be noted that some of the system answers might be valid and simple synonyms, even though they are not the same as proposed by the gold-standard corpus.

## Conclusions

Although DPLs should be designed and written ensuring complete understanding of their contents, several factors can have an influence on patient understanding of DPLs. Low literacy is directly associated with limited understanding and misinterpretation of these documents [45, 46]. Older people are more likely to have lower literacy skills, as well as decreased memory and poorer reading comprehension [47]. These factors may lead to an unintentional non-compliance or inappropriate use of drugs, leading to dangerous consequences for patients, such as therapeutic failure or adverse drug reactions.

Several studies [3, 4, 6] have shown that there is an urgent need to improve the quality of DPLs. In particular, patients have problems to understand those sections describing dosages and adverse drug reactions. Our work aims the simplification of DPLs, in particular, the substitution of terms describing drug effects by synonyms that are easier to understand by patients.

In our previous work, MedDRA and MedlinePlus were used as sources of synonyms for the drug effects in DPLs. Moreover, an index was built from a large collection of articles gathered from the MedlinePlus website. This index provided us information about how common a word is. This system proposed the most frequent synonym candidate because complex words tend to have lower frequency than simpler ones. This approach is considered to be baseline system. Experiments showed an accuracy of 68.7% for the MedlinePlus synonym and 37.1% for the MedDRA synonym. Therefore, we can conclude that resources that were specially written for patients are a better source of simpler synonyms than other specialized resources such as MedDRA.

To date, word embeddings have hardly ever explored for lexical simplification [31, 48]. In a word embedding model, each word in a corpus is represented with a vector in a semantic space. An important advantage of our approach is that it does not require terminological resources or manually simplified corpora (such as Simple Wikipedia^12^), which are expensive and time consuming to build and are not available for a vast number of languages and domains. In this work, we develop a system based on word embeddings, and our experiment results show an accuracy of 0.365%. However, as our approach employs a word embedding model to obtain the set of candidate synonyms, its main limitation is that it cannot propose multi-word synonyms. To overcome this problem, we gathered a set of phrase candidates from our collection of MedLilePlus articles by using a set of patterns based on POS tags. Then, we obtain their phrase embbeddings by averaging the word embeddings of their content words. Then, we calculate not only the similarity between words, but also with phrases. This approach achieves an improvement of 2% over the word embedding approach and of 1% over the baseline system using the MedDRA dictionary.

An error analysis shows that some of the system answers might be valid and simple synonyms, even though they are not the same as proposed by the gold-standard corpus. In order to obtain a more realistic evaluation, we plan to extend the EasyDPL corpus by adding several simpler synonyms for each term.

Although the results are lower than those achieved by the previous work using the MedlinePlus dictionary, we think that the approach is promising because it is possible to simplify DPLs without using terminological resources or parallel corpora. Therefore, our approach can be applied to different domains and languages. Thus, we plan to extend our approach in order to simplify not only other medical concepts (such as diseases, medical procedures, medical tests, etc), but also complex words from open-domain texts. As future work, we also plan to integrate additional resources such as BabelNet [49] or the UMLS Metathesaurus [50]. In addition to providing broader coverage for terms and more synonyms, these resources will allow to develop a multilingual simplification system. Moreover, we would like to extend the collection of texts used to train the word and phrase embedding model by adding texts directly related to the pharmacovigilance literature. We also plan to study how to improve our phrase embedding model to propose multi-words candidates as synonyms.

## Endnotes

^1^ https://www.ethnologue.com/statistics/size

^2^ http://www.cs.york.ac.uk/semeval-2012/task1/

^3^ http://easy-to-read.eu/

^4^ http://openoffice-es.sourceforge.net/thesaurus/

^5^ https://catalog.ldc.upenn.edu/LDC2011T07

^6^ https://www.aemps.gob.es/cima/inicial.do

^7^ https://www.nlm.nih.gov/medlineplus/spanish/

^8^ http://www.vademecum.es

^9^ https://www.prospectos.net

^10^ http://www.meddra.org/

^11^ http://www.nltk.org

^12^ https://simple.wikipedia.org

## Abbreviations

DPL: Drug package leaflets
E2R: Easy-to-read
NLP: Natural language processing
POS: Part-of-speech
